# Injury surveillance and associations with socioeconomic status indicators among youth/young workers in New Jersey secondary schools

**DOI:** 10.1186/s12940-016-0118-z

**Published:** 2016-02-16

**Authors:** Alexsandra A. Apostolico, Derek G. Shendell

**Affiliations:** Rutgers School of Public Health (SPH), Center for School and Community-Based Research and Education (CSCBRE), 335 George Street - Suite 2200, New Brunswick, NJ 08903 USA; New Jersey Safe Schools Program, 683 Hoes Lane West, 3rd Floor, Piscataway, NJ 08854 USA; Rutgers SPH, Department of Environmental and Occupational Health, 683 Hoes Lane West, 3rd Floor SPH Building, Piscataway, NJ 08854 USA; Rutgers University, Environmental and Occupational Health Sciences Institute-Exposure Measurement and Assessment Division, 170 Frelinghuysen Road, Piscataway, NJ 08854 USA

**Keywords:** Injury, Socioeconomic status, Young workers

## Abstract

**Background:**

Injuries involving career-technical-vocational education (CTE) are reported to the New Jersey Safe Schools Program online reporting system, the only U.S. State law-based surveillance data for young workers (ages twenty-one and younger), a susceptible, vulnerable adolescent sub-population.

**Methods:**

We examined potential associations between socioeconomic status (SES) indicators and high school student injuries reported between 12/1998-12/2013, excluding injuries acquired by staff members. Associations between DFG score—a proxy for school/district SES—and variables relating to reported injuries, including severity, injury type, injury cause, body parts injured, injury treatment setting and demographics were examined with chi square test (X^2^) for independence and logistic regression. To assess potential associations between SES and personal protective equipment (PPE), data were stratified by 2003–2008 and 2008–2013, given mandated payment by employers of PPE for employees.

**Results:**

Statistically significant associations were found between SES and injury cause [X^2^ = (7, 14.74), *p* = 0.04] and SES and injury treatment setting [X^2^ = (1, 4.76), *p* = 0.03]. Adjusted odds ratio suggested students from low SES schools were at a higher odds of being treated at a hospital emergency department (ED) than students from high SES schools (95 % CI 1.3–4.3, *p* < 0.01).

**Conclusions:**

These findings indicated low SES schools/districts have increased odds of being treated at ED, after controlling for injury severity. Future research should focus on implications such associations have on health care access and insurance for young workers and their families. With small sample sizes representing lower DFG scoring (SES) schools/districts, additional efforts should be enacted to increase injury reporting in these schools/districts.

## Background

Unintentional injuries among adolescents are a public health concern for several reasons, including the health impacts on an already susceptible, vulnerable subpopulation (twenty-one and younger) and the multifaceted issues leading to these unintentional injuries. One particular area of concern are injuries involving students in supervised, school-sponsored career-technical-vocational education (CTE) programs, and how socioeconomic status (SES) may relate to the occurrence of these injuries. Currently, there is a wealth of information supporting the association between SES and unintentional injuries among adolescents involving motor vehicle-related injuries, recreational-related injuries, falls, and sports-related injuries [1–4]; however, little is known about the association between SES and work-related injuries in adolescents, specifically injuries among students enrolled in CTE programs. Current literature has suggested an inverse association between SES and prevalence of adolescent work-related injuries, where lower SES is associated with a higher prevalence of adolescent work related injuries [5]. Research also has suggested SES influenced how young adults were treated in the work place by employers (e.g., being assigned dangerous tasks and a lack of overall supervision), which can have a direct impact on work place injuries [6, 7].

It has been estimated 70–80 % of teens work at some point during their high school career [8]. Because of their inexperience, and in many instances, their lack of knowledge concerning safety and health (S&H) topics, young workers have about twice the risk of incurring job-related injuries compared to older adults [8–12]. Other factors leading to higher rates of injury among young workers included a lack of physical and/or emotional maturity, lack of proper safety training, being unfamiliar with standard operating procedures for certain tasks, and how young workers maybe not be aware of legal limitations imposed on workers under age 18, including prohibited tasks and prohibited equipment under child labor laws [8, 13, 14]. The highest rates of work-related injuries have occurred in the 18–24 year old bracket, followed by 15–17 year olds [15]. It has been estimated in the United States (U.S.) each year about 160,000 young workers experience occupational related injury or illness; however, the National Institute of Occupational Safety and Health acknowledged how these numbers are widely underestimated and approximately two-third of work-related incidents are unreported [15].

CTE programs offer a great opportunity to prepare students to enter the work force. It has been estimated there are over 20,000 CTE vocational and ready to work programs in the U.S. [16]. Students in CTE programs are more likely to report having received safety training and having been informed of their legal rights compared to teens working outside of these structured programs [17, 18]. The U.S. Office of Vocational and Adult Education estimated, on average, every high school student has taken at least one CTE course, and 1-in-4 students have taken three or more courses in a single program area [16]. Clearly, CTE programs encompass a large percentage of young adults. Understanding injuries related to these programs and their association with SES is of public health significance.

Past evidence suggested children of families in the lowest income bracket have the highest rates of unintentional, nonfatal injuries; it is unclear, however, if this association holds true for adolescents enrolled in structured work programs, such as CTE programs [19]. Reducing the prevalence of work related injuries in this subpopulation would improve their S&H and also decrease medical expenditures, as unintentional injuries are a prominent source of medical spending for youth aged 21 years and younger in the U.S. [19].

SES is generally acknowledged as an important predictor of health status [6, 20–22]. Proxies of SES utilized throughout the literature usually encompassed some form of measurement of finance, education, and occupation. SES as it relates to chronic disease [20, 23] mental health, [24, 25] and infectious disease [26, 27] is relatively well studied; however, further investigation is warranted to examine potential associations between SES and unintentional injuries among young workers. Moreover, a literature review performed by Cubbin et al. in 2002 reported SES was an important predictor of injury; nevertheless, the direction and strength of this association depended on indicators used to measure SES [28–30].

The New Jersey (NJ) Department of Education, based on NJ Administrative Code 6A:19–6.5, requires by law for accidents/incidents (injury or illness) involving CTE students and/or staff that are treated by a licensed physician, physician’s assistant, or advanced practice nurse to be reported to the NJ Commissioner of Education [31–33]. These incidents are directly reported to the NJ Safe Schools Program (NJSS) online surveillance system (via Psychdata) for aggregate analyses. Data from submitted individual injury reports are collected on several key variables including reporting county, school district, and school name. Information is also collected on the gender; whether the injured person was a staff or student; the title of the co-op/structured learning experience program; and where the injured person was treated (doctor/clinic versus hospital/emergency department (ED)). Moreover, parts of body injured, nature of injury, cause of injury, severity of injury, use of personal protective equipment (PPE), and date and time of the injury are recorded [31–33].

Here, we examined potential associations between SES indicators and work-related injuries among adolescents, specifically injuries involving CTE programs in the state of NJ, excluding injuries acquired by school staff members. We hypothesized SES would be associated with several key variables examined including injury cause, injury location on the body, injury type, injury severity, use of PPE, where an injury was treated and gender of the injured individual. Specifically, we predicted individuals in lower SES schools/school districts would have more severe injuries, use PPE less often, or use PPE incorrectly compared to higher SES schools/school districts, and have an increased odds of being treated at a hospital. To test this hypothesis, we utilized data collected through the NJSS incident reporting surveillance system between the years 1998–2013 and District Factor Groups (DFG) scores, a proxy of SES [34].

## Methods

This data analysis represents aggregate injury surveillance data; no personal, identifying information was utilized. The Rutgers University-New Brunswick Institutional Review Board human subjects approved protocol number is 021997W0383.

In NJ, DFG scores are a proxy used to estimate a community’s relative SES, as calculated using six different variables to estimate SES. The variables are: percent of adults with no high school diploma, percent of adults with some high school education, occupational status, unemployment rate, percent of individuals in poverty, and median family income. Data for these variables were collected from the most recent U.S. Decennial Census.

Statistical analyses were conducted within SAS 9.4 (Cary, North Carolina). In order to explore associations between school-district SES indicators and CTE related injuries reported to NJSS, DFG scores were used as a proxy measurement of SES for each reporting school/school district. Schools were given a DFG score ranging from 1 (lowest score/lowest SES) through 8 (highest score/highest SES). From the DFG scores, schools were dichotomized into lower scoring (DFG scores 1–4) and higher scoring (DFG scores 5–8) schools. The NJ Department of Education does not assign DFG Scores to county vocational school districts. Therefore, DFG scores were summed and averaged across the county as a whole, and the mean county score was used to assess SES for each reporting school in that county. Descriptive analyses were then performed to describe the demographics of the overall study population, as well as the study population stratified by DFG score. DFG was stratified in two ways, both as raw scores, ranging from 3–6 (for our study population) and as a dichotomous variable either being low (3 and 4) or high (5 and 6). This dichotomous classification coincides with low SES and high SES, respectively. Chi square tests (Χ^2^) for independence were conducted in order to examine associations between SES (high versus low) and various variables including gender, injury-treatment setting (hospital versus doctor/clinic), injury location on body, injury type, injury cause, severity of injury, and use of PPE, as these variables were hypothesized to be related to SES. Logistic regression was then conducted to further explore associations between SES and injury report variables listed above. Both crude and adjusted models were explored. The adjusted models included several variables hypothesized to potentially confound the association between SES and injury treatment setting. These variables were severity of injury, injury type, body part injured, and injury cause. We further hypothesized how the severity of injury would be the most important predictor of where an injury would be treated, as severity would determine if an injury needed acute attention from the ED, or further care that could be given at a doctor’s office; therefore, a preliminary model adjusted solely for severity of injury was examined. To be conservative in the interpretation of results, the final model included adjustments for each of these variables.

To assess potential associations between SES and PPE use, data were stratified by the years 2003–2008 and 2008–2013. This stratification of years was chosen because starting in 2008 employers were legally required to pay for properly selected and fitted PPE determined necessary for employees (NJ as of 2/2008, U.S. as of 10/1/2008) [35]. PPE usage data were also stratified on career cluster, categorized as either being hazardous or nonhazardous. Categorization of career clusters as hazardous or non-hazardous was based on the 17 non-agricultural hazardous occupations orders (HOs) and 11 agricultural HOs of the U.S. Department of Labor (U.S. DOL) [36]. Based on this classification there were eight career clusters categorized as hazardous: (1) agriculture, food, and natural resources; (2) architecture and construction; (3) transportation, distribution, and logistics; (4) manufacturing; (5) law, public safety, corrections, and security; (6) human services (e.g. cosmetology programs); (7) health sciences; and, (8) science, technology, engineering, and mathematics. Eight career clusters were thus categorized as non-hazardous: (1) marketing; (2) arts, audio/video technology, and communication; (3) business management and administration; (4) education and training; (5) finance; (6) hospitality and tourism; (7) government and public administration; and, (8) information and technology. It should be noted this classification scheme excluded the fact there are hazards present in each career cluster, for example, ergonomic factors which are accounted for in every workplace/workstation environment, including those career clusters classified as non-hazardous.

## Results and discussion

The twenty-one counties in NJ had at least one school district submit an injury report between 1998 and 2013. Sixteen counties were classified as higher DFG scoring schools/districts; nine scored a “6”, and seven scored a “5”. Five counties were classified as lower DFG scoring schools/districts; four scored a “4” and one scored a “3”. Demographic results of the study population are described in Table 1. Overall, there was an even distribution of where an injury was treated, with 56 % being treated at hospitals and 44 % at doctors/clinics. The most common body part injured were fingers, making up 38 % of reported injuries. The most common type of injury was cut/laceration (43 %). The most common injury cause was ‘struck by’ (35 %). Overall, most injuries reported were non-disabling (68 %); 32 % of injuries were temporarily disabling and there was only one reported permanently disabling injury. In this context, the term temporarily disabling meant the student was able to return to their school-sponsored SLE after medical treatment and rest. Overall, the use of PPE was fairly evenly split—47 % of reported injuries stated some kind of PPE was in use at the time of the incident. A majority or 95 % of reporting schools were classified as high SES (scores 5 and 6), and 5 % were classified as low SES (scores 3 and 4).

**Table 1:** Summary of descriptive statistics for variables on injury reports to NJ Safe Schools state-law based surveillance, 12/1998-12/2013

Characteristic	Total (*n*)	Total (%)
DFG		
3	8	0.4
4	87	4.5
5	755	39.0
6	1088	56.1
DFG Score		
High	1847	95.3
Low	91	4.7
Gender		
Male	1373	71.7
Female	543	28.3
Status		
Student	1809	96.4
Staff	69	3.7
Treatment		
Hospital	746	55.6
Doctor	595	44.4
Injury Location		
Finger	722	38.1
Hand	199	10.5
Eye	143	7.6
Foot	68	3.6
Back	50	2.6
Face	45	2.4
Other	281	14.9
Multiple	188	9.9
Arm	63	3.3
Head	53	2.8
Knee	33	1.7
Ankle	49	2.6
Injury Type		
Fracture	70	4.0
Burn	171	9.7
Bruise/Bump	60	3.4
Sprain	90	5.1
Puncture	71	4.0
Other	344	19.6
Multiple	150	8.5
Abrasion	45	2.6
Cut/Laceration	758	43.1
Injury Mode		
Struck Against	292	15.9
Caught In/Under/Between	113	6.2
Extreme Temperature	145	7.9
Other	451	24.6
Rubbed/Abraded	47	2.6
Fall (Same Level)	86	4.7
Struck By	641	35.0
Multiple	57	3.1
Severity		
Non-disabling	1230	68.1
Temporary disabling	575	31.9
Permanent Disability	1	0.1
PPE		
No	230	47.2
Yes	258	52.9

When stratifying by SES (high versus low), the distribution of gender remained similar to the ratio in the overall study population. Males consisted of 72 % of the sample population in high SES schools, and 65 % in low SES schools. When stratified by SES, where injuries were treated differed between high and low SES schools (*p* = 0.029). For high SES schools, location of where injuries were treated was fairly evenly distributed—55 % were treated at hospitals and 45 % were treated by doctors/clinics. On the other hand, in low SES schools, 68 % of injuries were treated at hospitals, while only 32 % were treated by doctors/clinics. Severity of injury did not change substantially when stratified by SES (*p* = 0.87). In high SES schools, 68 % of injuries were non-disabling and 32 % were temporarily disabling. The one reported injury resulting in a permanent disability during the study period, an amputation of fingers, was reported in a high SES school. For low SES schools, 70 % of reported injuries were non-disabling and 30 % were temporarily disabling. For both high and low SES schools, finger was the most common body part injured, ‘struck by’ the most common cause of injury, and cut/laceration the most common injury type.

Distribution of injury reports and use of PPE were analyzed based on the time period in which they were reported (2003–2008 vs. 2008–2013), as well as whether the injury reported was associated with a hazardous or non-hazardous career cluster (Table 2). We were interested in comparing PPE use in high SES and low SES schools. Table 2 depicts results from the PPE use analysis. We observed a trend, where, in general, reported incidents with a response of “Yes” to PPE use decreased from the first time block (2003–2008) to the second time block (2008–2013). And, this trend was observed in general, regardless of hazardous and SES classifications. For example, those reporting “Yes” to PPE use in high SES schools in hazardous career clusters decreased from 76 % to 61 % (71 incidents to 62 incidents) between the two time blocks. An exception to this trend included those incidents among students in low SES schools/districts that reported “Yes” to PPE use in non-hazardous career clusters; however, low cell sizes should be considered. We observed no consistent trend for those who reported “No” for PPE use. In general, the number of incidents in which it was reported “No” for PPE use increased from the first time block (2003–2008) to the second time block (2008–2013). For example, those reporting “No” for PPE use in high SES schools in hazardous career clusters increased from 24 % to 39 % (22 incidents to 39 incidents) between the two time blocks. We also observed those schools/districts who reported “No” PPE use in low SES schools in hazardous career clusters remained stable, though cell sizes were low. It should also be noted those who reported “No” PPE use in low SES schools in non-hazardous career clusters decreased by half (from six incidents to three incidents) even if cell sizes were low between years. In summary, as schools/districts which reported using PPE reported fewer injuries than schools/districts which reported not using PPE, these results suggested schools/districts using PPE are selecting properly fitting PPE and using PPE correctly at a higher rate. Further research is needed for more definitive conclusions on the impact of the state/federal laws requiring purchase of PPE by employers for employees and of specific PPE training. Increase reporting of injuries among low SES schools would also strengthen conclusions.

**Table 2:** Injury incidents by occupation type and PPE use: New Jersey Safe Schools surveillance data (12/1998 through 12/2013)

			03/2003-08/2008					9/2008-10/2013				
PPE usage		Overall injury reports 2003–2013 (N = 393)	High SES		Low SES			High SES		Low SES		
			Hazardous	Non-hazardous	Hazardous	Non-hazardous	Sum	Hazardous	Non-hazardous	Hazardous	Non-hazardous	Sum
Yes	N	214	71	49	3	0	*123*	62	24	2	3	91
	(%)	54.0	76.3	59.8	60.0	0.0		61.4	25.0	50.0	50.0	
		Within Time Period (%)	57.7	39.8	2.4	0	100.0	68.1	26.4	2.2	3.3	100.0
No	N	179	22	33	2	6	63	39	72	2	3	116
	(%)	46.0	23.7	40.2	40.0	100.0		38.6	75.0	50.0	50.0	
		Within Time Period (%)	34.9	52.4	3.2	9.5	100.0	33.6	62.1	1.7	2.6	100.0

Note: Totals include injury reports indicating both use of PPE and a career cluster

Descriptive analysis was carried out to better understand associations between SES and injury reports. Chi square test (Χ^2^) for independence (Table 3) revealed statistically significant associations between SES and injury cause [X^2^ = (7, 14.74), *p* = 0.04] as well as SES and injury treatment setting [X^2^ = (1, 4.76), *p* = 0.03]. A series of logistic regressions were performed to further examine potential associations between SES and the binary outcome injury treatment setting. To understand the potential differences in treatment locations within each SES group (high versus low), logistic regression was performed comparing the location of treatment in the high vs. low SES schools. Unadjusted odds ratios (OR) suggested low SES schools had an increased odds of being treated at a hospital/ED compared to high SES schools (OR = 1.75; 95 % CI = 1.1–2.9; Table 4). Similarly, when adjusting for severity of injury, low SES schools still had an increased odds of being treated at a hospital/ED compared to high SES schools (adjusted odds ratio [AOR] = 1.80; 95 % CI = 1.2–1.9; Table 4). Further, when adjusting for severity of injury, injury type, body part injured and injury cause, low SES schools had an increased odds of being treated at a hospital/ED compared to high SES schools (AOR = 2.40; 95 % CI = 1.3–4.3; Table 4).

**Table 3:** High versus low SES schools/districts: injury reports to New Jersey Safe Schools state-law based surveillance, 12/1998-12/2013

Characteristic	High SESn (%)	Low SES n (%)	p-value
Gender			
Male	1,315 (72.0)	58 (65.2)	0.160
Female	512 (28.0)	31 (34.8)	
Treatment			
Hospital	697 (54.9)	49 (68.1)	0.029
Doctor	572 (45.1)	23 (31.9)	
Injured Body Part			
Finger	675 (37.4)	47 (53.4)	0.140
Hand	191 (10.6)	8 (9.1)	
Multiple	180 (10.0)	8 (9.1)	
Arm	61 (3.4)	2 (2.3)	
Head	53 (2.9)	0 (0.0)	
Knee	31 (1.7)	2 (2.3)	
Ankle	48 (2.7)	1 (1.1)	
Eye	136 (7.5)	7 (8.0)	
Foot	68 (3.8)	0 (0.0)	
Back	48 (2.7)	2 (2.3)	
Face	42 (2.3)	3 (3.4)	
Other	273 (15.1)	8 (9.1)	
Injury Type			
Fracture	64 (3.8)	6 (6.8)	0.481
Burn	162 (9.7)	9 (10.2)	
Bruise/Bump	58 (3.5)	2 (2.3)	
Sprain	85 (5.1)	5 (5.7)	
Puncture	64 (3.8)	7 (8.0)	
Multiple	141 (8.4)	9 (10.2)	
Abrasion	43 (2.6)	2 (2.3)	
Cut/Laceration	724 (43.4)	34 (38.6)	
Other	330 (19.7)	14 (15.9)	
Injury Cause			
Struck Against	281 (16.1)	11 (12.6)	0.039
Caught In /Under/Between	100 (5.7)	13 (14.9)	
Extreme Temperature	137 (7.9)	8 (9.2)	
Rubbed/Abraded	45 (2.6)	2 (2.3)	
Fall (Same Level)	80 (4.6)	6 (6.9)	
Struck By	617 (35.4)	24 (27.6)	
Multiple	54 (3.1)	3 (3.4)	
Other	431 (24.7)	20 (23.0)	
Severity			
Non-Disabling	1,168 (68.0)	62 (70.5)	0.870
Temporary Disabling	549 (32.0)	26 (29.5)	
Permanently Disabling	1 (0.10)	0 (0.0)	
PPE			
Yes	246 (53.2)	15 (55.6)	0.400
No	216 (46.8)	12 (44.4)

**Table 4:** Odds ratio (OR) for being treated at a hospital emergency department

Characteristic	OR	95 % CI	*p*-value
SES^a^			
High	Referent		
Low	1.75	1.1–2.9	0.03
SES^b^			
High	Referent		
Low	1.8	1.1–3.1	0.02
SES^c^			
High	Referent		
Low	2.4	1.3–4.3	<0.01

^a^Crude OR

^b^Adjusted for severity of injury

^c^Adjusted for severity of injury, injury type, body part injured, and injury cause

Previous literature has suggested childhood injuries varied by SES, especially relating to morbidity and mortality [22]. The goal of the present analysis was to better understand potential associations between SES indicators and reported CTE-related injuries. Data reported here represents surveillance data captured between 12/1998 through 12/2013 using the NJSS law-based CTE reporting system, aggregated through Psychdata. We hypothesized SES status would be associated with several key variables including injury cause, injury location on the body, and injury type, injury severity, use of PPE, and where an injury was treated. Specifically, we hypothesized the low SES schools/districts would have more severe injuries, use PPE less often and/or use PPE incorrectly, compared to high SES schools/districts, and have an increased odds of being treated at a hospital/ED.

Statistically significant differences were observed between SES and injury cause. These results suggest a statistically significant association between SES and CTE related injuries and where injuries are treated. For high SES schools, the distribution of injury treatment setting was fairly evenly distributed, with 55 % being treated at hospitals and 45 % being treated by doctors/clinics. In low SES schools, however, a greater difference was observed, with 68 % of injuries being treated at hospitals/ED and 32 % being treated by doctors. Our logistic regression analyses (both crude and adjusted) supported this observation, suggesting low SES schools/districts have increased odds of being treated at a hospital/ED compared to high SES schools/districts. Thus, an argument can be made for the discrepancy between injury treatment setting and SES being in part an issue of access, both in a physical sense (e.g., lack of private/public transportation given distance to travel) and in regards to health care (e.g., number of providers) and/or health care insurance. It should be noted how these data cannot fully capture the recent changes in health care reform, i.e., the passing of the Patient Protection and Affordable Care Act (ACA) in 2010 and its implementation ongoing in 2013 through the present [37]. Future research with NJSS surveillance data may better capture changes in health care status in relation to the ACA. Further research should also be conducted to explain why these discrepancies existed. Current literature has suggested poor individuals who are underinsured or uninsured typically seek care in hospital/ED [38]. It should be noted, however, as these injuries were acquired within school sponsored CTE programs, cost incurred would potentially be covered by the reporting school/district. Although this may suggest how health insurance status does not play as vital role in the injury treatment setting of a reported injury, individuals often times do not know how to navigate the health care system, or lack the knowledge concerning reimbursement by reporting school/district. Irrespective of who is responsible for paying for treatment, the cost to society as a whole is vast, with each trip to an ED expecting to cost around $200.00 [39]. Moreover, links have been made between workplace S&H and income inequality by the U.S. DOL, which stated workplace injuries may have a greater effect on low wage workers and those trying to enter the middle class. Similarly, U.S. DOL has noted workplace injuries (examined in the adult population) placed burdens on the workers and their families and have contributed to income inequality [40]. Further research may examine how outcomes of workplace injuries affect varying SES grades differently. This analysis explores the association of income inequality and workplace S&H as it related to students participating in school-sponsored CTE programs. It should also be considered how effect modification may be at play here, skewing the relationship between workplace injury outcomes and SES [41]. There are also areas of improvements regarding the data sources used. The NJ Department of Education should strive to provide direct DFG scores for vocational-CTE schools. A limitation of this study was NJ DFG scores were not available for the twenty-one reporting county-based CTE schools/districts. To overcome this limitation, DFG scores were summed and averaged across reporting schools in each county. These county-average DFG scores were used for each reporting school in each respective county. This limitation may have led to non-differential misclassification bias of exposure—whether the school was reported as a low SES school or a high SES school—and a bias towards the null, i.e., underestimating the true association between SES and where incidents were treated (hospital/ED versus doctor). Also limiting current analysis was the lower number incident reports from low SES schools compared to high SES schools due to the small number of counties classified as low SES schools. In total, there were 95 injury reports from low SES schools and 1,843 injury reports from high SES schools. Future efforts should be placed on increasing injury reporting in low SES schools.

Another limitation of this study was there were no denominator data—data were reported incidents within CTE programs. There was no information concerning students/staff enrolled in CTE programs that were not injured between the years 1998–2013. Another limitation related to the generalizability of results to the general secondary school/student population. Students enrolled in NJ CTE programs represent a growing yet specific subset of the overall student population. These students may not be generalizable to the general student body throughout NJ, and may not be generalizable to other state CTE students, as CTE programs differ state by state. Another limitation, as is true with most surveillance data, was missing and incomplete data fields. However, as of October 2013, reports are only submitted online to NJSS via Psychdata. This eliminates the ability to leave certain spaces blank as occurred with the past paper based system. Future analyses will compare completeness of reporting factors between the former paper-based and current online reporting system.

This study also had several strengths. This study represented surveillance data over fourteen years for the entire state of New Jersey. These findings add to a major gap in the literature by specifically examining injuries relating to secondary school students enrolled in CTE programs by SES. This study also identified areas to be further analyzed in order to (1) reduce the rates of injures for secondary school students enrolled in CTE programs; (2) reduce total medical expenditures resulting from CTE related injuries; and, (3) improve the overall quality of life for secondary school students enrolled in CTE programs.

## Conclusion

Initial findings from this current analysis suggested there is a statistical difference between high and low SES schools and injury cause, as well as high and low SES schools and injury treatment setting. Results from this analysis suggested injuries occurring in low SES schools have higher odds of being treated at a hospital compared to an injury reported at a high SES school. This association remained true after for controlling for several key variables including injury severity. Future research should explore why this may be the case, and better assess whether this is an issue of medical access, and/or school policy as to where an injury is initially treated. This study’s results can guide future development of injury prevention trainings and interventions, which can lead to decreased rates of injuries, decreased medical expenditures, and increased student academic performance and achievements.

## Abbreviations

ACA: Patient Protection and Affordable Care Act
AOR: adjusted odds ratio
CI: confidence interval
CTE: Career and Technical Education
DFG: District Factor Group
DOL: Department of Labor
ED: emergency department/emergency room
HOs: hazardous occupations orders
NJ: New Jersey
NJ SS: NJ Safe Schools Program
OR: odds ratio
PPE: personal protective equipment
S&H: safety and health
SES: socioeconomic status
SLE: school-sponsored structured learning experiences
U.S.: United States
